# CovidVisualized: Visualized compilation of international updated models’ estimates of COVID-19 pandemic at global and country levels

**DOI:** 10.1186/s13104-022-06020-4

**Published:** 2022-04-09

**Authors:** Farshad Pourmalek

**Affiliations:** 1 University of British Columbia, Vancouver, Canada; 2 1604-9541 Erickson Dr, Burnaby, BC V3J 7N8 Canada

**Keywords:** COVID-19, Pandemic, Epidemic, Models, Visualization, Global, Canada, Iran

## Abstract

**Objectives:**

To identify international and periodically updated models of the COVID-19 epidemic, compile and visualize their estimation results at the global, regional, and country levels, and periodically update the compilations. This compilation can serve as an early warning mechanism for countries about future surges in cases and deaths. When one or more models predict an increase in daily cases or infections and deaths in the next one to three months, technical advisors to the national and subnational decision-makers can consider this early alarm for assessment and suggestion of augmentation of preventive measures and interventions.

**Data description:**

Five international and periodically updated models of the COVID-19 pandemic were identified, created by: (1) Massachusetts Institute of Technology, Cambridge, (2) Institute for Health Metrics and Evaluation, Seattle, (3) Imperial College, London, (4) Los Alamos National Laboratories, Los Alamos, and (5) University of Southern California, Los Angeles. Estimates of these five identified models were gathered, combined, and graphed at global and two country levels. Canada and Iran were chosen as countries with and without subnational estimates, respectively. Compilations of results are periodically updated. Three Github repositories were created that contain the codes and results, i.e., “CovidVisualizedGlobal” for the global and regional levels, “CovidVisualizedCountry” for a country with subnational estimates–Canada, and “covir2” for a country without subnational estimates–Iran.

## Objective

### Objectives and rationale

The objectives are to identify international and periodically updated models of the COVID-19 epidemic, compile and visualize their estimations’ results at the global and country levels, and periodically update the compilations. The ultimate objective is to provide an early warning system for technical advisors to the decision-makers. When the predictions of one or more models show an increase in daily cases or infections, hospitalizations, or deaths in the next 1–3 months, technical advisors to the national and subnational decision-makers may consider assessing the situation and suggesting augmentation of non-pharmacologic preventive interventions and vaccinations. No similar work provides visualization of the models’ results in one place and keeps records of the previous updates. This paper describes why and how the CovidVisualized tools were created and how countries can use them. It is possible to create and use such an early warning tool for future surges in the pandemic in a way that is usable by researchers and the technical advisers to policymakers.

### Eligibility criteria

The criteria for inclusion of target COVID-19 models were (1) an international model scope and (2) periodic updates. “International model” denotes a model that estimates COVID-19 cases or infections and deaths for all countries of the world, with global-level estimates that equate the sum of the national-level estimates. “Periodically updated” denotes a model with a record of periodically updated estimates since its first release, with continued updates in 2021.

### Finding the eligible models

The eligible models were found within the literature search of a previous publication, “Rapid review of COVID-19 epidemic estimation studies for Iran” [1]. The results were verified by comparison with models found in a recently published study on “Predictive performance of international COVID-19 mortality forecasting models” [2].

### Identified eligible models

Five international and periodically updated models of the COVID-19 pandemic were identified: (1) DELPHI,^1^ Massachusetts Institute of Technology, Cambridge (abbreviation used in this work: DELP) [3], (2) Institute for Health Metrics and Evaluation, Seattle (IHME) [4], (3) Imperial College, London (IMPE) [5], (4) Los Alamos National Laboratories, Los Alamos (LANL) [6], (5) University of Southern California, Los Angeles, by Srivastava, Ajitesh (SRIV) [7].

## Data description

### Repositories for codes and data sharing

Three Github repositories were created for this project: “CovidVisualizedGlobal” [8] for the global and regional levels, “CovidVisualizedCountry” [9] for countries with subnational estimates, and “covir2” [10] for countries without subnational estimates. Canada and Iran were chosen for case representation of each of the two types of countries, respectively (see Table 1). These are referred to as CovidVisualized GitHub repositories hereon.^2^ Six World Health Organization regions were used for the regional level: African Region (AFR), Americas Region (AMR), Eastern Mediterranean Region (EMR), European Region (EUR), South-East Asian Region (SEAR), and Western Pacific Region (WPR).

**Table 1:** Overview of data files/data sets

Label	Name of data file/data set	File types (file extension)	Data repository and identifier (DOI or accession number)
Data set 1	CovidVisualizedGlobal, COVID-19 estimates at the global level	Stata code (.do), log (.smcl), data (.dta); R code (.R); data (.csv), graph (.pdf)	Zenodo repository http://doi.org/10.5281/zenodo.5019030 [8]
Data set 2	CovidVisualizedCountry, COVID-19 estimates at the country level: Canada	Stata code (.do), log (.smcl), data (.dta); R code (.R); data (.csv), graph (.pdf)	Zenodo repository http://doi.org/10.5281/zenodo.5019482 [9]
Data set 3	covir2, COVID-19 estimates at the country level: Iran	Stata code (.do), log (.smcl), data (.dta); R code (.R); data (.csv), graph (.pdf)	Zenodo repository http://doi.org/10.5281/zenodo.5020797 [10]
Data set 4	CovidVisualized Methodology Document	Word, PDF	Zenodo repository http://doi.org/10.5281/zenodo.6371475 [11]

### Data management

A template was created to assign comparable variable names to various outcomes from different models. The CovidVisualized methodology document explains the conceptual and computational details of the development of CovidVisualized tools and provides examples[11].^3^ Stata SE 14.2 (Stata Statistical Software. StataCorp. College Station, Texas) was used to write and run the codes. Graphs for all types of predicted outcomes, their mean estimates and uncertainty limits, and different scenarios within each model where available are created. IHME and IMPE models have alternative (e.g., “better” and “worse) scenarios besides their reference (aka status quo) scenario. Predictions’ graphs are shown on the pages of the three CovidVisualized GitHub repositories [8–10] and in periodical Situation Reports created with each uptake. The DELP and IHME models provide subnational-level estimates for countries reporting national and subnational level COVID-19 outcomes. Graphs were created for national and subnational-level locations (i.e., provinces in Canada) available in DELP and IHME model outputs.

### Periodical uptakes

The two models with the least frequency of periodic updates of estimates are IHME and IMPE, updated almost weekly and bi-weekly, respectively–until November 2021. After the spread of the Omicron variants, these models reduced the frequency of their update releases. Therefore, two sets of arrangements ruled the frequency of performing uptakes in the CovidVisualized tools. The first set covered the year 2021: with the release of each update of either of these two models, the whole set of the five included models are updated in all the three CovidVisualized GitHub repositories. The most recent update of each model is used. The conventions for periodical uptake are described in detail in CovidVisualized methodology document [11]. R software via RStudio 1.4 (Integrated Development for R. RStudio. PBC, Boston, Massachusetts) was used for semi-automatization of the uptakes’ execution. Estimates of the LANL model get updated about every 3–4 days, and DELP and SRIV models get updated daily. The second set of arrangements for the frequency of performing uptakes in the CovidVisualized tools started in 2022. Uptakes are conducted each week on Friday. Each uptake uses the latest available update of each model.

### Similar work

The “covidcompare” tool [12] provides graph visualization of the latest estimates of daily and total deaths from international and periodically updated COVID-19 models for countries of the world and US states, along with historical forecasts and model performance, based on IHME’s “Predictive performance of international COVID-19 mortality forecasting models” [2].

## Limitations

### Limitations

Stata programming language constitutes about 99% of the codes. Whereas Stata is a commercial software package, using non-commercial packages such as R and/or Python can increase the accessibility and adaptability of the codes for other researchers. Further use of R and/or Python can also make the uptakes almost fully automatized. Some health researchers may not be familiar with GitHub and GIT programming. Therefore, additional use of a dedicated website that is more visible to and accessible for the target audience can increase the reach and effect of this work. Strengths and weaknesses of individual international and periodically updated COVID-19 pandemic models are not mentioned here, but they have been discussed elsewhere [1, 2] and in the CovidVisualized Methodology Document [11].

## Data Availability

The data described in this Data Note can be freely and openly accessed on (1) GitHub repository “CovidVisualizedGlobal” under (http://doi.org/10.5281/zenodo.5019030) [8], (2) GitHub repository “CovidVisualizedCountry” under (http://doi.org/10.5281/zenodo.5019482) [9], and (3) GitHub repository “covir2” under (http://doi.org/10.5281/zenodo.5020797) [10]. Please see Table 1 and references [8–11] for details and links to the data. No individual patient data was mentioned to be used for modeling in the five models used in this work [13–18]. Third-party data has been used in this study and their relevant attributions are available [19–24] and observed.

## Abbreviations

AFR: African Region (World Health Organization)
AMR: Americas Region (World Health Organization)
CoMo (Consortium): COVID-19 International Modelling Consortium
CovidVisualizedCountry: Covid Visualized Country. A GitHub repository that was created in this work for visualization of COVID-19 epidemic models’ estimates at country level—countries with subnational estimates, e.g., Canada
CovidVisualizedGlobal: Covid Visualized Global. A GitHub repository that was created in this work for visualization of COVID-19 epidemic models’ estimates at global level
covir2: COVID Iran Review Number 2. A GitHub repository that was created in this work for visualization of COVID-19 epidemic models’ estimates at country level—countries without subnational estimates, e.g., Iran
DELP: DELPHI. Differential Equations Lead to Predictions of Hospitalizations and Infections. COVID-19 pandemic model named DELPHI developed by Massachusetts Institute of Technology, Cambridge
EMR: Eastern Mediterranean Region (World Health Organization)
EUR: European Region (World Health Organization
IHME: Institute for Health Metrics and Evaluation COVID-19 pandemic model by developed Institute for Health Metrics and Evaluation, Seattle.
IMPE: Imperial COVID-19 pandemic model developed by Imperial College, London.
LANL: Los Alamos National Laboratories COVID-19 pandemic model developed by Los Alamos National Laboratories, Los Alamos.
SEAR: South-East Asian Region (World Health Organization).
SRIV: Srivastava, Ajitesh. COVID-19 pandemic model developed by Ajitesh Srivastava, University of Southern California, Los Angeles.
WHO: World Health Organization.
WPR: Western Pacific Region (World Health Organization)

## Footnotes

1 DELPHI: Differential Equations Lead to Predictions of Hospitalizations and Infections (model).

2 Web site addresses for these repositories are https://github.com/pourmalek/CovidVisualizedGlobal, https://github.com/pourmalek/CovidVisualizedCountry, and https://github.com/pourmalek/covir2 respectively.

3 Web site address for the CovidVisualized methodology document is https://github.com/pourmalek/CovidVisualizedMethodology.
